# Hedonic Capacity in the Broader Autism Phenotype: Should Social Anhedonia Be Considered a Characteristic Feature?

**DOI:** 10.3389/fpsyg.2016.00666

**Published:** 2016-05-06

**Authors:** Derek M. Novacek, Diane C. Gooding, Madeline J. Pflum

**Affiliations:** ^1^Department of Psychology, Emory UniversityAtlanta, GA, USA; ^2^Department of Psychology, University of Wisconsin–MadisonMadison, WI, USA; ^3^Department of Psychiatry, University of Wisconsin–MadisonMadison, WI, USA

**Keywords:** pleasure, social interaction, hedonic capacity, autism, ACIPS, TEPS

## Abstract

Impairments in social motivational processes may partially explain the differences in social interaction seen among individuals with autism spectrum disorder (ASD). The social motivation hypothesis would predict an association between reduced hedonic capacity and ASD. However, to date, findings have been mixed regarding hedonic deficits among individuals with ASD; adults report lower levels of both social and physical pleasure whereas adolescents only report experiencing lower social pleasure. Moreover, very few studies examining the association between anhedonia and autistic traits have used measures of hedonic response or taken temporal aspects of pleasure into account. The present study examined associations between autistic traits and the experience of pleasure using a non-clinical sample of young adults to further clarify the nature of hedonic deficits in the broader autism phenotype (BAP). Results revealed that autistic traits were negatively associated with both the experience of social pleasure as well as general pleasure, although the association was stronger for social pleasure. Regression analyses revealed that reduced social pleasure was a better predictor of autistic traits than general pleasure. Together these findings suggest that reduced social hedonic capacity is associated with autistic traits in the general population and should be included in conceptualizations of the BAP.

## Introduction

Autism spectrum disorders (ASD) are phenomenologically and etiologically heterogeneous neurodevelopmental disorders characterized by social, communicative, and cognitive impairments as well as repetitive and restrictive behaviors or interests (American Psychiatric Association, 2013). Research suggests that from early on, individuals with ASD differ from typically developing individuals. For example, rather than orienting to biological motion, toddlers with ASD prefer non-social contingencies (Klin et al., 2009). In an auditory attention task, preschool children with ASD preferred non-speech signals to motherese speech (Kuhl et al., 2005). Moreover, both children and adults with ASD show increased preference for objects instead of people in social scenes (Klin et al., 2002). These early social deficits often persist throughout development, resulting in impairments in more complex social cognitive processes such as Theory of Mind (ToM; Baron-Cohen et al., 1985; Williams and Happé, 2010), social perception (Klin et al., 2002), and emotion recognition (Balconi and Carrera, 2007; Kennedy and Adolphs, 2012). Numerous theories have been proposed to explain these features of the autism spectrum. More recently, the social motivation theory has gained empirical support as a partial explanation for these differences (Chevallier et al., 2012b).

The social motivation theory of autism posits that deficits in motivational processes constitute a primary impairment in ASD (Chevallier et al., 2012b). Early-onset deficits in social attention contribute to developmental processes that disrupt social learning, cognition, and skill development. These deficits in social attention are thought to reflect reduced social interest and motivation. Thus, the theory asserts that because individuals with ASD do not find social interactions rewarding, they are not interested in pursuing these interactions. Reduced social attention and low interest/motivation over time impairs social cognitive abilities due to inadequate frequency of learning opportunities. Within this framework, research has demonstrated that social orienting, social seeking and liking, as well as social maintaining are all impaired in individuals with ASD (see Chevallier et al., 2012b, for review).

In addition to the behavioral studies that have demonstrated findings consistent with the social motivation theory, some research findings provide evidence that the autism spectrum is characterized by a different response toward socially salient rewards compared to non-social rewards. Using monetary and social incentive delay tasks, individuals with ASD showed decreased activation of the dorsal striatum compared to controls in response to social rewards, but not to monetary rewards, suggesting discrepant responses contingent on the type of reward (Delmonte et al., 2012). When participating in two versions of a rewarded implicit learning task, children with ASD displayed diminished neural responses to both social and monetary rewards, with a more pronounced reduction in response to social rewards, particularly in frontostriatal regions compared to typically developing (TD) children (Scott-Van Zeeland et al., 2010). Moreover, adults with ASD showed atypical amygdala and insular cortex activation while processing social rewards (Dichter et al., 2012). Conversely, other neuroimaging studies found evidence of a general reward dysfunction rather than a deficit specific to social reward (Kohls et al., 2013). However, these inconsistent findings may be due to a lack of ecologically valid social reward paradigms. In a novel behavioral paradigm, namely, the choose-a-movie task (Dubey et al., 2015), investigators provided some intriguing findings. First, they observed that among TD adults, there is a reliable preference for social (smiling faces directly gazing at one) stimuli rather than non-social stimuli (either averted gaze or object). A second study by the same group revealed that adults with ASD showed less preference for the social stimuli (i.e., direct gaze faces) than the TD adults; these findings are consistent with the social motivation hypothesis of autism. In contrast, children and adolescents with ASD did not display any differences in the value placed on social stimuli in a behavioral econometric choice task (Watson et al., 2015). However, the children and adolescents with ASD placed more value on non-social images related to restricted interests (e.g., trains and electronics). These mixed findings highlight the need for further research to clarify the nature of reward-related differences associated with the autism spectrum.

Hedonic capacity is a component of the reward processing system that also includes motivation (i.e., wanting or incentive salience) and learning (Berridge and Robinson, 2003). Although hedonic response is a crucial component in our understanding of the possible reward-related processes that underlie social deficits, thus far relatively few studies have examined hedonic response among individuals with ASD.

In their comparison of adolescents with and without ASD, Chevallier et al. (2012a) found that the groups did not differ in terms of their self-reported experience of physical pleasure, though they reported experiencing significantly less pleasure from social situations. These findings suggest that ASD may be associated with a selective hedonic deficit in the social domain (i.e., social anhedonia). In contrast, Berthoz et al. (2013) found that adults with ASD endorsed both greater physical and social anhedonia than controls. Clearly, there is a need for further research to clarify the nature of hedonic deficits in the autism spectrum.

Most research that has examined hedonic deficits in the autism spectrum has done so by examining associations between autistic traits and measures of negative schizotypy such as the Schizotypal Personality Questionnaire (Raine, 1991) and the Oxford-Liverpool Inventory of Feelings and Experiences (Mason et al., 1995). These studies have consistently shown positive associations between autistic traits and anhedonia as a feature of schizotypy in both clinical (Spek and Wouters, 2010) and non-clinical populations (Rawlings and Locarnini, 2008; Claridge and McDonald, 2009; Russell-Smith et al., 2011).

However, little is known about associations between autism spectrum traits and measures of hedonic response that are not particular to schizophrenia. One study that investigated the association between autistic traits and hedonic response was conducted by Foulkes et al. (2015). In their online investigation of adults aged 18–65 (mean age of 35), Foulkes et al. (2014) examined associations between autistic traits and several aspects of social reward using the Social Reward Questionnaire. The SRQ assesses six unique domains of social reward, including sociability, the enjoyment of group interactions. Results revealed a negative association between autistic traits and sociability, which was not accounted for by alexithymia, another construct measured in their study. Overall, few studies have examined the specific nature of social/interpersonal pleasure in relation to autism spectrum traits, particularly using validated measures of hedonic capacity. Furthermore, the measures used in previous studies of autistic traits only assessed social anhedonia and did not differentiate the anticipatory and consummatory components of the experience of pleasure.

In the past few decades, a significant amount of research has focused on examining the autism spectrum below the clinical threshold. With the dimensional nature of autistic traits, the broader autism phenotype (BAP) refers to the presence of autistic characteristics in the general population, including siblings and other relatives of individuals diagnosed with ASD (Piven, 2001; Sucksmith et al., 2011). Investigations into the nature of hedonic deficits within the BAP using non-clinical samples could provide evidence for whether or not reduced hedonic capacity is associated with risk for ASD.

Although autistic traits are thought to be distributed continuously in the general population (Constantino and Todd, 2003; Ruzich et al., 2015), social anhedonia appears to be follow a more skewed distribution in the normal population, so that it is a relatively uncommon trait. Thus, it is beneficial to examine the association between autistic traits and social anhedonia in a non-clinical sample. One of the advantages of studying this association in a non-clinical sample is that one can avoid the possible confounds associated with the disorder, such as impaired communication. Another advantage is that by investigating the association between autistic traits and social anhedonia in a non-clinical sample, any correlation may be less likely to be attributable to atypical social experiences resulting from having a developmental disorder, possibly being placed in different educational settings and/or different social contexts such as group homes. In this way, studying the association between autistic traits and social anhedonia in TD individuals may enable us to gain insights regarding whether lack of enjoyment of social/interpersonal interactions is secondary to lack of exposure to certain types of interactions, reflective of social skills deficits, or more related to an attentional difficulty, broadly defined. Finally, taking a dimensional approach allows us examine whether there is a threshold effect, i.e., identify whether a certain level of functioning is necessary in order for individuals to possibly benefit from certain psychosocial interventions intended to ameliorate social anhedonia.

The aim of the present study was to examine relations between autism spectrum traits and the self-reported experience of pleasure to provide further support for the social motivation theory of autism using validated measures of hedonic capacity, namely the Anticipatory and Consummatory Interpersonal Pleasure Scale (ACIPS; Gooding and Pflum, 2011, 2014b) and the Temporal Experience of Pleasure Scale (Gard et al., 2006). Moreover, we sought to further clarify the nature of this association by using measures that not only discriminate between social and general pleasure, but also the anticipatory and consummatory aspects of the experience of pleasure. We hypothesized that autism spectrum traits would be negatively associated with both social and general pleasure. We also hypothesized that lower scores on a measure of social pleasure would be a stronger predictor of autism spectrum traits than lower scores of general pleasure. Consistent with previous studies (Gard et al., 2006; Gooding and Pflum, 2014a), we expected females to report experiencing more social as well as general pleasure than males.

## Materials and Methods

### Participants

This was a non-clinical sample of English speaking undergraduate students at a large Midwestern university. The participants are part of a larger ongoing research endeavor. Two-hundred and sixty-five students enrolled in introductory psychology courses over six semesters were recruited to participate and complete a battery of questionnaires which included the measures in the present study. No information was gathered regarding the psychiatric history of the participants.

### Measures

#### Autism Spectrum Quotient (AQ)

The Autism Spectrum Quotient (AQ; Baron-Cohen et al., 2001) is a self-report measure designed to assess autism spectrum traits in individuals from the general population with average or above IQs. The AQ consists of 50 items, each rated on a 4-point Likert-based scale, ranging from “strongly disagree” to “strongly agree.” The responses are scored dichotomously, whereby an endorsement in the direction of the autistic trait (either mildly or strongly) is scored “+1” and the opposite response is scored as a “0.” The maximum score on the AQ is 50, with higher scores indicating the presence of more autistic traits. Baron-Cohen et al. (2001) suggested that a score of 32 or higher on the AQ indicated the presence of an ASD. Subsequently, classifications based upon AQ scores became more fine-grained. Wheelwright et al. (2010) operationally defined BAP based upon AQ scores, whereby the BAP is 1 to 2 SDs above the mean (AQ between 23 and 28), the medium autism phenotype is used to describe AQ scores that are 2 to 3 Ss above the mean (i.e., AQ between 29 and 35) and individuals classified in the “narrow autism phenotype” have scores falling 3 SDs or above the mean (i.e., AQ scores greater than 35). The total score has been shown to have moderate to high internal consistency with coefficient alphas ranging from 0.67 to 0.82 (Austin, 2005; Hurst et al., 2007). In the present sample, the AQ demonstrated moderate internal consistency (coefficient α = 0.73). The total AQ score as well as the three-factors proposed by Austin (2005) were used to examine associations with hedonic capacity. This includes a social skills factor, a details and patterns factor, and a communication/mind reading factor. In the factor analysis conducted by Austin (2005), some items did not load on a factor and were left out.

#### Anticipatory and Consummatory Interpersonal Pleasure Scale (ACIPS)

The ACIPS is a 17-item self-report measure designed to assess one’s capacity to experience pleasure in the interpersonal and social domains (Gooding and Pflum, 2011, 2014b). Items are scored on a 6-point Likert scale, ranging from 1 (very false for me) to 6 (very true for me), with lower scores indicating social anhedonia, or reduction in the capacity to experience interpersonal pleasure. Gooding and Pflum (2014a) showed that the items load onto four factors: (1) general social interactions, (2) close relationships, (3) bonding over shared interests and experiences, and (4) family-related interactions. The psychometric properties of the ACIPS have been described elsewhere. Briefly, the ACIPS has high internal consistency, temporal stability, convergent, and discriminative validity (Gooding and Pflum, 2014a,b; Gooding et al., 2015). In the present sample, the ACIPS demonstrated high internal consistency (coefficient α = 0.90).

#### Temporal Experience of Pleasure Scale (TEPS)

The TEPS is an 18-item self-report measure used to assess individual experiences of anticipatory and consummatory pleasure (Gard et al., 2006). Items are rated on a scale of 1 (very false for me) to 6 (very true for me), with 10 of them assessing anticipatory and eight assessing consummatory pleasure. Because most of the items on the TEPS assess non-social pleasure, it was included to be a comparison of the ACIPS as a measure of more general pleasure. The TEPS has been shown to have good internal consistency and test–retest reliability (Gard et al., 2006). In the present sample, the TEPS demonstrated good internal consistency (coefficient α = 0.80).

### Statistical Analyses

Although the TEPS and ACIPS were administered in one document, the items were separated and the two measures were scored separately. Linear regression was used to examine hedonic response as a predictor of autistic traits. Bivariate correlations were used to examine associations between scales. An alpha of *p* < 0.001 determined significance after applying a Bonferroni correction to account for multiple correlations. When comparing associations between the two scales with the AQ, Steiger’s r-to-z transformation was used (Steiger, 1980). Independent samples *t*-tests were used to examine possible sex differences. All *p*-values are two-tailed. All data preparation and analyses were performed using SPSS version 21.

### Procedure

The present study was approved by the University of Wisconsin–Madison Social and Behavioral Sciences Institutional Review Board. Written informed consent was obtained before the study began. All participants were at least 18 years of age. Participants were administered a battery of questionnaires (the “Mass Survey”) during class. The total administration time was 30 min. Participants received extra credit points in exchange for their participation.

## Results

**Table 1** provides detailed demographic description of the sample. Briefly, the sample consisted of 265 students (162 males, 103 females) with a mean age of 18.95 (±1.44) years. Consistent with the racial and ethnic makeup of the undergraduate population, 81.5% of the sample identified as Caucasian.

**Table 1:** Demographic characteristics and descriptive statistics for the self-report measures.

Variable	Entire Sample (*n* = 265)	Males (*n* = 162)	Females (*n* = 103)
Age^a^	18.95 ± 1.44	19.02 ± 1.62	18.85 ± 1.09
Age range	18–28 years	18–28 years	18–23 years
Racial/Ethic Background^b^			
Caucasian	216 (81.5)	139 (85.8)	77 (74.8)
Black/African– American	7 (2.6)	4 (2.5)	3 (2.9)
Asian	18 (6.8)	9 (5.6)	9 (8.7)
Hispanic/Latino	14 (5.3)	3 (1.9)	11 (10.7)
Other	10 (3.8)	7 (4.3)	3 (2.9)
AQ total^a^	17.83 ± 5.74	18.07 ± 5.86	17.45 ± 5.55
Autism phenotype^bc^			
Typical	210 (79.3)	127 (78.4)	83 (80.6)
Broader	43 (16.2)	26 (16.0)	17 (16.5)
Medium	12 (4.5)	9 (5.6)	3 (2.9)
Narrow	0 (0)	0 (0)	0 (0)
ACIPS total^a^	84.35 ± 11.61	82.59 ± 11.51	87.13 ± 11.28
TEPS^a^			
Total^a^	77.26 ± 10.14	74.86 ± 9.86	81.05 ± 9.44
Anticipatory	39.80 ± 5.28	39.92 ± 5.22	41.19 ± 5.10
Consummatory	37.46 ± 6.53	35.94 ± 6.71	39.85 ± 5.48

*^a^Mean ± standard deviations are provided. ^b^Frequency and (percentage) are provided. Descriptive statistics are provided for the following self-report measures: the Baron-Cohen Autism Spectrum Quotient (AQ; Baron-Cohen et al., 2001); Anticipatory and Consummatory Interpersonal Pleasure Scale (ACIPS; Gooding and Pflum, 2011, 2014b); and the Temporal Experience of Pleasure (TEPS; Gard et al., 2006). ^c^The autism phenotype classification was made based upon the total AQ scores, with cutoffs operationally defined by Wheelwright et al. (2010).*

### Autism Spectrum Traits and Hedonic Capacity

**Table 1** also provides descriptive statistics for each of the self-report measures. Participants were grouped according to the different autism phenotypes, based upon AQ totals, with cutoffs operationally defined by Wheelwright et al. (2010). Although none of the sample met criteria for the narrow autism phenotype, approximately 20% of the participants fell into either the broader or medium autism spectrum phenotype category.

**Table 2** provides the Pearson correlation coefficients for associations between autism spectrum traits and various aspects of pleasure. Autism spectrum traits, as measured by the AQ total score, were negatively associated with the experience of social/interpersonal pleasure as measured by the ACIPS total score, *r* = -0.47 *p* < 0.001. Autism spectrum traits were also significantly and inversely associated with the four empirically derived factor loadings from the ACIPS, namely: general social interactions (*r* = -0.47), close relationships (*r* = -0.37), bonding over shared interests and experiences (*r* = -0.32), and family-related interactions (*r* = -0.24), *p*s < 0.001, respectively. When we examined the AQ factor scores and the ACIPS scores (see **Table 2**), the only AQ factor scores that were significantly associated with the ACIPS total score was the AQ social factor (*r* = -0.63, *p* < 0.001). Autism spectrum traits were also negatively associated with TEPS anticipatory pleasure (*r* = -0.31, *p* < 0.001) and TEPS consummatory pleasure (*r* = -0.30, *p* < 0.001). Steiger’s r-to-z transformation revealed a significant difference in the strength of the correlations between autism spectrum traits and the two measures of pleasure. The association between autism spectrum traits and social/interpersonal pleasure was significantly greater than the association between those traits and general pleasure, *z* = -2.49, *p* < 0.01. There was no difference in the strength of the association between AQ scores and anticipatory versus consummatory pleasure, *z* = -0.17, *p* = 0.87.

**Table 2:** Relationships between autistic traits and aspects of pleasure.

Variable	1	2	3	4	5	6	7	8	9	10	11	12
(1) AQ Social												
(2) AQ Details	0.14											
(3) AQ Comm.	0.16	0.13										
(4) AQ Total	0.74*	0.52*	0.52*									
(5) ACIPS I	-0.61*	-0.10	-0.10	-0.47*								
(6) ACIPS II	-0.52*	-0.06	0.02	-0.37*	0.76*							
(7) ACIPS III	-0.50*	-0.01	-0.03	-0.32*	0.57*	0.51*						
(8) ACIPS IV	-0.31*	-0.14	-0.01	-0.24	0.34*	0.36*	0.32*					
(9) ACIPS Total	-0.63*	-0.09	-0.05	-0.46*	0.93*	0.91*	0.70*	0.49*				
(10) Anticipatory	-0.50*	-0.07	0.01	-0.31*	0.55*	0.63*	0.49*	0.25*	0.64*			
(11) Consummatory	-0.36	-0.04	-0.06	-0.30*	0.44*	0.57*	0.57*	0.16	0.54*	0.47*		
(12) TEPS Total	-0.47*	-0.06	-0.04	-0.35*	0.57*	0.70*	0.70*	0.23	0.68*	0.82*	0.89*	

Correlation matrix depicting bivariate correlations between autism spectrum traits, as measured by the Autism Spectrum Quotient (AQ; Baron-Cohen et al., 2001). AQ factors based upon Austin (2005) are provided: Social, Details (and Patterns), and Comm(unication). The experience of pleasure was measured by the ACIPS (Gooding and Pflum, 2014b) and the TEPS (Gard et al., 2006). Four ACIPS Factor scores are provided: Factor I: General Social Interactions; Factor II: Close Relationships; Factor III: Bonding over Shared Interests and Experiences; and Factor IV: Family-related Interactions). The two TEPS subscale scores, namely, Anticipatory Pleasure and Consummatory Pleasure, are provided as well. *p < 0.001.

In order to determine whether correlations between the ACIPS and AQ were due to items assessing social pleasure in the AQ, items that referred to the enjoyment of social interactions were removed from the social factor proposed by Austin (2005). This included items #17 (“I enjoy social chit-chat”), 44 (“I enjoy social occasions”), and 47 (“I enjoy meeting new people”). After removing the three aforementioned items, the association between the ACIPS total score and the AQ social factor remained significant (*r* = -0.59, *p* < 0.001), suggesting that these items were not solely driving the association.

We performed linear regression analyses to examine whether social pleasure as measured by the ACIPS was a significant predictor of autism spectrum traits. The results revealed that the ACIPS total score was a significant predictor of autism spectrum traits, β = -0.462, *p* < 0.001, accounting for 21% of the variance. When the TEPS anticipatory and consummatory scores were added as predictors in the second block, the ACIPS remained a significant predictor, β = -0.420, *p* < 0.001. However, the TEPS anticipatory and consummatory scores were not significant predictors of autism spectrum traits, β = -0.013, *p* = 0.86 and β = -0.062, *p* = 0.35, respectively.

In a subsequent model in which the TEPS scores were entered first, the model was significant, indicating that the TEPS anticipatory and consummatory scores were significant predictors of autism spectrum traits, β = -0.223, *p* < 0.01 and β = -0.190, *p* < 0.01, respectively. However, when the ACIPS was added in the second block, the TEPS anticipatory and consummatory scores were no longer significant predictors, β = -0.013, *p* = 0.86 and β = -0.062, *p* = 0.35, respectively. However, the ACIPS total score was a significant predictor, β = -0.420, *p* < 0.001.

### Gender Differences in Self-report Measures

The male and female respondents did not differ in terms of their level of endorsement of autism spectrum traits, *t*(263) = 0.87, *p* = 0.39. However, we observed a gender difference in terms of total ACIPS scores, with the female participants reporting significantly higher levels of social pleasure, *t*(263) = 3.15, *p* < 0.01. Similarly, we found that females reported both more anticipatory pleasure [*t*(263) = 3.488, *p* < 0.001] and consummatory pleasure [*t*(263) = 4.97, *p* < 0.001], as measured by the TEPS, than males.

## Discussion

The results from the present study suggest that non-clinical individuals with a greater number of autism spectrum traits, as measured by the Autism Spectrum Quotient, are likely to report lower levels of social and interpersonal pleasure, as measured by the ACIPS. The higher the presence of autism spectrum traits, the less pleasure participants reported experiencing from social and interpersonal contexts. This relationship held constant even after removing AQ items that referred to the enjoyment of social interactions from the social factor proposed by Austin (2005).

In this investigation, autism spectrum traits were negatively associated with hedonic capacity. However, we noted that the relationship between autism spectrum traits and pleasure for social interactions, as measured by the ACIPS, was significantly stronger than the relationship between autism spectrum traits and general pleasure, as measured by the TEPS. This relationship was confirmed by regression analyses, which indicated that social pleasure was a better predictor of autism spectrum traits than the experience of general pleasure. Indeed, although TEPS scores predicted autistic traits, this effect was not significant when ACIPS scores were introduced into the model.

These results are consistent with two previous studies that examined the nature of hedonic capacity using clinical samples of individuals with ASD, in which the ASD groups reported experiencing lower levels of social pleasure than controls (Chevallier et al., 2012a; Berthoz et al., 2013). Although both social and general pleasure were negatively associated with autistic traits, the stronger association with social pleasure suggests that reduced social hedonic capacity may be particularly characteristic of the autism spectrum. Given that hedonic capacity and motivation are related components of reward processing, these findings can be viewed as further support for the social motivation theory of autism, which asserts that deficits in social motivational mechanisms contribute to the social deficits experienced by individuals with ASD (Chevallier et al., 2012b). Our present findings appear wholly consistent with a recent report by Foulkes et al. (2015), in which non-clinical adults displayed negative inverse relationships between autistic traits and reported levels of enjoyment of prosocial interactions (“sociability”) as measured by the Social Reward Questionnaire.

Although prior work has demonstrated that individuals ASD report experiencing lower levels of social pleasure than TD individuals, it was important to examine whether the association between autistic traits and diminished social interest would be evident in a non-clinical sample using a modern, validated measure of social/interpersonal hedonic pleasure. The results of the present study can be viewed as further evidence for social anhedonia as a core feature of the BAP, a term used to refer to the presence of subthreshold autistic features and traits in individuals who do not meet criteria for ASD (Piven, 2001).

While the present findings appear consistent with the social motivation theory, there are equally plausible alternative accounts for the association between reduced hedonic capacity and autism spectrum traits. An example of such an alternative is the view that the social anhedonia observed among ASD individuals is secondary to their ToM deficits. According to Krach et al. (2010), mental processing and interpretation of the mental states of one’s social interaction partners is what gives the social interaction its rewarding quality. Hence, according to this view, it is a ToM impairment that renders an individual less able to find social interactions pleasurable, rather than the individual necessarily having less motivation to engage in social interactions *per se*. Indeed, there seems to be some support for this view, given increasing numbers of reports of loneliness among college-aged and older adults with ASD (see, for example, Jobe and White, 2007). Viewed either way, these findings have treatment implications. Decreased social pleasure has been associated with higher levels of self-reported loneliness and social interaction anxiety, two constructs related to other forms of psychopathology (Gooding et al., 2015). Thus, individuals who endorse higher levels of autism spectrum traits who also report social anhedonia may benefit from psychotherapy focused on improving their social functioning and/or decreasing any anxiety related to social interactions.

## Limitations, Strengths, And Future Directions

All of the participants in the present study were college undergraduates. Use of a non-clinical sample therefore limits the external validity of the study findings to community samples of educated young adults. Indeed, no one in the present sample met criteria, based on the threshold suggested by the developers of the AQ, for the presence of an ASD and very few met criteria for even the medium autism phenotype. We have taken care to speak about autism spectrum traits. Research based upon a clinical sample (i.e., individuals diagnosed with ASD) that asks similar questions as those addressed here would be imperative to further address the nature of the relationship between social hedonic capacity and autism spectrum traits. Thus, we are hopeful that this study will serve as a heuristic for future research in this regard.

Another limitation of the present study is our failure to include a measure of alexithymia. Prince and Berenbaum (1993) observed that alexithymia is significantly associated with social anhedonia. In prior research from our lab (Gooding and Tallent, 2003), we observed that 23% (10 of 43) of our socially anhedonic participants were identified as alexithymic on the basis of their elevated scores on the Toronto Alexithymia Scale (TAS-20; Bagby et al., 1994). Prior research indicates that there is a strong and inverse relationship between the Chapman revised Social Anhedonia Scale and the ACIPS (Gooding and Pflum, 2014a,b; Gooding et al., 2015). Moreover, studies of individuals with and without ASD have indicated that alexithymia predicted reduced empathic brain activation (Bird et al., 2010), as well as poor recognition of emotional facial expressions (Cook et al., 2013). Taken together, these facts might lead one to question whether the negative association between autistic traits and hedonic capacity might be accounted for by the elevated levels of alexithymia that may co-occur in individuals with a greater number of autistic traits. While this is a plausible hypothesis, we think it is a less tenable one, given the results of the Foulkes et al. (2015) investigation. In the Foulkes et al. (2015) study, although there was an association between autistic traits, social reward, and alexithymia, alexithymia did not contribute unique variance to the prediction of sociability, enjoyment, or admiration, or prosocial interactions. Prior research has demonstrated positive and significant associations between ACIPS total scores and SRQ Admiration, Prosocial Interaction, and Sociability scores (Gooding et al., 2015). Thus, one cannot conclude that alexithymia can account for the association between autistic traits and hedonic capacity. Further research using measures of hedonic capacity such as the ACIPS is needed in order to more robustly test the relationships between the constructs of alexithymia, capacity for social/interpersonal pleasure, and autistic traits.

While previous studies examining relations between autistic traits and anhedonia yielded consistent results (Rawlings and Locarnini, 2008; Claridge and McDonald, 2009; Berthoz et al., 2013), further exploration was warranted given that most of the earlier studies used measures of schizotypy to assess anhedonia rather than measures of hedonic response. One strength of the present study is that we included two distinct, well-validated measures of pleasure. In this way, we were able to distinguish hedonic capacity for social and interpersonal interactions from hedonic capacity for general stimuli and situations. Moreover, we were also able to distinguish the temporal aspects of hedonic response (i.e., anticipatory versus consummatory pleasure) in the relation with autistic traits. Future research would be enhanced by the inclusion of behavioral paradigms for assessing social motivation, such as the recently developed choose-a-movie paradigm (Dubey et al., 2015). This would be useful in terms of an increased focus on identifying the mechanisms underlying social anhedonia in ASD individuals. Ideally, a multi-method investigation would allow researchers to study pleasure and social interactions while distinguishing social skills from hedonic capacity. It would also be interesting to examine how amenable self-reported social anhedonia is in individuals with various levels of autism spectrum traits.

## Conclusion

The present study indicates that individuals with greater number of autism spectrum traits are more likely to report lower levels of social/interpersonal pleasure. These findings are consistent with growing evidence for social anhedonia being part of a BAP. It is critical that future work in this area include clinical samples in order to permit further exploration of the association between autism spectrum traits and social anhedonia. It is also imperative that future studies investigating the association between autism spectrum traits and social anhedonia use measures that specifically assess hedonic capacity for social and interpersonal stimuli and situations, as well as measures that are developmentally appropriate for the age of the sample.

## Author Contributions

DCG conceived of the project, designed the project, and revised the manuscript for important intellectual content. She is the primary developer of the ACIPS. DMN collected some of the data, analyzed the data, and prepared the first draft of the manuscript. MJP trained DMN, oversaw all of the data collection, and helped to critically revise the manuscript for important intellectual content. MJP is a co-developer of the ACIPS.

## Conflict of Interest Statement

The authors declare that the research was conducted in the absence of any commercial or financial relationships that could be construed as a potential conflict of interest.
